# Keeping the door open: virtual coverage of rural emergency departments—what works and what doesn’t

**DOI:** 10.1007/s43678-024-00855-5

**Published:** 2025-02-20

**Authors:** Brydon Blacklaws, John Pawlovich, Lauren Currie, Anurag Singh

**Affiliations:** 1 Department of Emergency Medicine, Powell River, BC Canada; 2 Rural Coordination Center of BC, Vancouver, BC Canada; 3 Department of Family Practice, University of British Columbia, Vancouver, BC Canada; 4 Northern Centre for Clinical Research (NCCR), University of Northern British Columbia, Prince George, BC Canada

**Keywords:** Rural health, Virtual care, Hybrid care, Health human resources, Physician burnout, Santé rurale, Soins virtuels, Soins hybrides, Ressources humaines en santé, Burnout médical

A growing number of rural communities are facing the nightmare of emergency department (ED) closures and diversions, with the nearest hospital often 2–3 h away. Mounting stories of adverse patient outcomes affecting rural and remote communities are morally distressing and hard to ignore. Despite the commitment of healthcare providers and organizations, we do not have enough physicians to work all the shifts in rural EDs. In times like these, communities often look to the Real-Time Virtual Support^1^ program in British Columbia (BC) to see if their site would work well with a virtual physician covering the vacant shifts as their ‘most responsible physician’ overnight.

Since 2021, the Real-Time Virtual Support Program has covered > 540 overnight single-coverage ED shifts, with a back-up community physician available to be called in if needed (Table 1). This has been a positive endeavour that has decreased rural ED diversion [1]. A virtual shift is covered by an experienced emergency medicine physician (EM) with virtual health training. Most shifts are 12 h, from 8 PM to 8 AM. The back-up physician is usually a family physician who has privileges at the local ED. The introduction of virtual physician coverage has prevented >6000 h of diversion and lessened the burden of overnight calls for rural doctors, which has been shown to reduce burnout while also supporting the recruitment and retention of physicians in rural areas [2].

**Table 1:** Number of shifts in rural BC communities supported by the virtual physician most responsible physician program since 2021 through December 2024

Communities	Population (2021)	Shifts completed
Fort Nelson	419	156
Nakusp	1589	86
Dawson Creek	12,323	40
Tumbler Ridge	2399	39
Lillooet	2302	39
Chetwynd	2302	47
Port McNeill	2356	30
Fort St James	1386	26
Burns Lake	1659	23
Mackenzie	3281	26
100 Mile	1928	18
Robson Valley	451	8
Vanderhoof	4346	8
Masset	838	6
Oliver	5094	4
Bella Coola	937	7

Despite benefits to rural EDs, clinicians and patients, the process has experienced challenges, and the following are some of the learnings and opinions we wish to share thus far.

## Optimal rural ED for virtual physician coverage

The optimal ED for virtual physician coverage would have one or fewer patient visits an hour on average, while the community overall has seven or fewer physicians. A busier site suffers some predictable issues, as discussed below.

## Which patients can be seen virtually?

This is generally a shared decision between the local clinician (e.g. rural nurse) and the virtual physician. Patients are often either ‘clearly appropriate’ or ‘clearly inappropriate’ for virtual care and it is easy for both virtual and local clinicians to agree. From a virtual physician perspective, patients can be sorted into four groups:

1. *The diagnosable*: the appropriate cases are those where a combination of the virtual physician assessment (via video), in-person registered nurse (RN) exam and investigations makes a diagnosis relatively easy and agreeable by the clinicians and patient. Examples include uncomplicated infections, mild injuries, flare ups of known conditions, and vague presentations seeking reassurance. Similar to circumstances in larger EDs, patients seen overnight—stable but not straightforward to diagnose—may wait for labs or imaging until the morning. These patients may either spend the night in the ED or return in the morning, giving the virtual physician comfort in knowing such patients will be reassessed in the morning. Abdominal or chest pain (not yet diagnosed) with routine workups, febrile kids without a clear source, and vague symptoms that are not easily explainable are all good examples.
2. *The clear admission*: some patients just are not going home. Whether they are ‘weak and dizzy’ with unremarkable initial work-up, alcohol withdrawal, mental health issues or acute medical conditions requiring admission, they can be safely worked-up and managed by an RN and virtual physician overnight. The virtual physician can write holding orders, and the local physician can admit the patient in the morning with their reassessment and exam.
3. *The clear transfer*: many transfers that require a ‘higher level of care’ can be managed by the virtual physician without involving the local physician. These patients are generally stable but need medical care beyond the rural hospital site's capacity or specialty care, such as internal medicine, orthopedics, or general surgery. Transfer services and accepting regional hospitals have been very supportive of virtual physician-initiated transfers without the local physician’s involvement, as they are aware of the staffing crises in rural sites.
4. *The procedural consult*: most patients needing a minor procedure are appropriate for a virtual physician. Virtual physicians can perform the majority of patient assessments, initiate workups and treatments, then rely on either the local RN or wait for the local physician to perform necessary procedures. For example, draining an abscess, reducing a wrist, or suturing a large laceration can be managed virtually with a ‘procedural consult’. In these cases, the local physician can simply assist procedurally when required, spending much less time than if they were solely responsible for the case.

## Challenges with virtual physician coverage

For larger rural communities with higher patient volume and acuity, a single coverage virtual physician and RN model faces some predictable challenges:

1. *Provider vulnerability*: when local nurses get too busy and stretched, valid concerns can be raised that reliance on a virtual physician decreases patient throughput, compared to an onsite physician. This can result in significant waits, high rates of leaving without being seen, and a feeling among staff that they are not practicing in a safe environment. Concerns like these can damage local provider morale and even cause nurses at larger rural sites to avoid shifts where only a virtual physician is covering.
2. *Loss of local back-up*: the safe practice of the virtual physician with a local RN scenario depends on a community physician coming in if needed. In larger rural sites, the problem is the local doctor gets called in almost every night—which is unsustainable when that physician has already worked all day and is expected back in the clinic/ED the next morning.
3. *Delayed procedures*: with higher patient volume/acuity comes a higher rate of procedures. Many procedures are not urgent but not ideal to be held all night. For example, open lacerations or wrist reductions should be done promptly to optimize healing and pain control. Trying to hold these procedures all night is not optimal, and the virtual physician often gets caught between wanting to call the local physician for the procedure and knowing that they might be depriving that clinician of their only opportunity for rest.
4. *Discontinuity of care*: patient care suffers when a virtual night shift is forced to hand over to a virtual day shift or a closed/diversion ED. A model where a virtual physician does not get the opportunity to have the local physician assess undifferentiated patients should be avoided at all costs.
5. *Day-time virtual coverage*: day shifts are almost always too busy for a virtual physician/RN team in a rural ED. This has been tried in multiple sites and should be avoided, unless the virtual physician is seeing patients alongside a local physician.

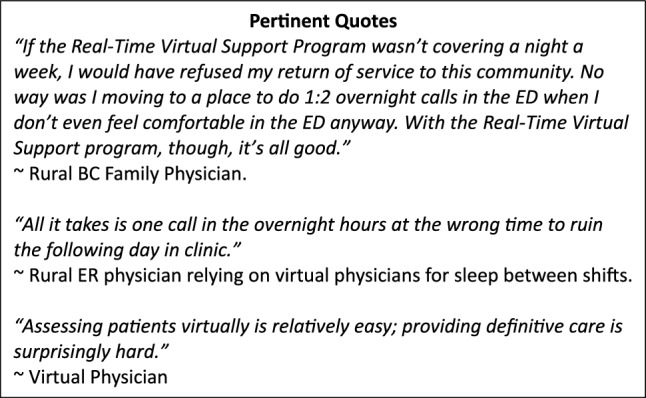


## The future of virtual most responsible physician care

Emerging evidence can be extrapolated to help rural EDs at risk of diversion.

1. *Virtual triage*: recent evidence suggests that virtual triage, whereby a patient presents to the ER and is ‘seen’ and assessed by a virtual physician, may reduce the burden on EDs in rural areas [3]. Similarly, virtual physicians can help with diversion support decisions post-triage, helping decide which patients can wait vs. those needing immediate transfer.
2. *Virtual buddy shifts*: supporting local providers, not just RNs, has great potential [4, 5]. Since 2020, the Real-Time Virtual Support program has paired a virtual physician with various clinicians, including: medical students, residents, community first responders, clinical associates, and even paramedics. Overall, this virtual buddy shift model is a functional way to improve the scope of practice of a local clinician and support a rural ED overnight.
3. *Team-based care*: a care model that includes a virtual physician, a local RN, and a local physician on duty has much potential, especially for medium-sized and urban EDs that want to add a virtual stream to their workflow. This model is most helpful when dedicated nursing staff are assigned to the virtual physician so that the local team does not deem the ‘inefficiency tax’ of the virtual physician too high.
4. *Nursing station support*: in BC, nursing stations are the point of care for many rural communities, and virtual physicians have been used to support RNs without local physician backup for decades. This model of care is an essential option for places that simply do not have a physician to be called in but cannot safely be put on diversion.

Additional research is needed to understand how best to implement this model for keeping EDs open, which communities are well served by this model, and how to ensure patient and provider safety. When any of us rush ourselves or a loved one to an ED in the middle of the night, we all want the same thing—a kind, welcoming, and experienced team of clinicians to help with our medical emergency. When that is not possible, a model that includes a virtual physician, with an onsite RN and local physician on-call has proved safe and effective in rural BC over the past four years [1]. Research on virtual support is just beginning, and we are excited to see more work around this innovative area in the years to come.

## Footnotes

1 The Real-Time Virtual Supports Program is the umbrella organization for multiple physician pathways. The Rural Urgent Doctor in Aid (RUDi) Program (45 + docs) is the emergency physician program, and the newest Real-Time Virtual Support Pathway, called VERRa (Virtual ER Rural Assist), is dedicated to the overnight coverage of rural Eds. To learn more, please see our website: https://rccbc.ca/initiatives/rtvs/
